# An electric field cell for performing *in situ* single-crystal synchrotron X-ray diffraction

**DOI:** 10.1107/S1600576721007469

**Published:** 2021-09-04

**Authors:** Lucy K. Saunders, Hamish H.-M. Yeung, Mark R. Warren, Peter Smith, Stuart Gurney, Stephen F. Dodsworth, Inigo J. Vitorica-Yrezabal, Adrian Wilcox, Paul V. Hathaway, Geoff Preece, Paul Roberts, Sarah A. Barnett, David R. Allan

**Affiliations:** a Physical Science, Diamond Light Source, Harwell Science and Innovation Campus, Didcot, Oxfordshire OX11 0DE, United Kingdom; b School of Chemistry, The University of Birmingham, Haworth Building, Edgbaston, Birmingham B15 2TT, United Kingdom; c Technical, Diamond Light Source, Harwell Science and Innovation Campus, Didcot, Oxfordshire OX11 0DE, United Kingdom; d Department of Chemistry, The University of Sheffield, Brook Hill, Sheffield S3 7HF, United Kingdom; e Department of Chemistry and Photon Science Institute, The University of Manchester, Oxford Road, Manchester M13 9PL, United Kingdom; f Life Science, Diamond Light Source, Harwell Science and Innovation Campus, Didcot, Oxfordshire OX11 0DE, United Kingdom

**Keywords:** electric field crystallography, synchrotron instrumentation, proton transfer

## Abstract

With the recent increase in research into ferroelectric, anti-ferroelectric and piezoelectric materials, studying the solid-state properties *in situ* under applied electric fields is vital in understanding the underlying processes. Where this behaviour is the result of atomic displacements, crystallographic insight has an important role. This work presents a sample environment designed to apply an electric field to single-crystal samples *in situ* on the small-molecule single-crystal diffraction beamline I19, Diamond Light Source (UK). The configuration and operation of the cell is described as well as its application to studies of a proton-transfer colour-change material.

## Abstract 2

This paper describes the design and implementation of an electric field sample cell, used to perform *in situ* single-crystal synchrotron X-ray diffraction under an applied electric field and suitable for single-crystal samples that are greater than 100 µm in size.

## 1. Introduction   

Solid-state materials can exhibit interesting dielectric phenomena on the application of an electric field. The range of behaviours includes ferroelectricity, where a spontaneous and switchable polarization is exhibited in (typically) polar systems under an electric field (Horiuchi *et al.*, 2012 ▸); anti-ferroelectricity, where symmetry-opposed polar sub-units present in a material may be aligned on application of an electric field and can be coupled with a crystallographic phase transition (Tolédano & Guennou, 2016 ▸); piezoelectricity, where the system shows a mechanical response to the field with change in, for example, the lattice/structural parameters (Werling *et al.*, 2013 ▸); proton-transfer behaviour (Rode *et al.*, 2016 ▸; Horiuchi *et al.*, 2008 ▸); and the enhancement of nonlinear optical properties (Bai *et al.*, 2013 ▸). A range of materials exist that exhibit electric field responses in the solid state, including metal oxides, metal–organic frameworks (Zhang & Xiong, 2012 ▸), hydrogen-bonded organic molecular crystals (Stroppa *et al.*, 2011 ▸; Horiuchi & Tokura, 2008 ▸; Owczarek *et al.*, 2016 ▸; Horiuchi *et al.*, 2020 ▸) and ionic solids (Li *et al.*, 2015 ▸; Schmalzried & Smolin, 1998 ▸; Zhang *et al.*, 2018 ▸; Rodzevich *et al.*, 2017 ▸). Related to their dielectric properties, these materials can have applications as pressure sensors (Haertling, 1999 ▸), actuators (Wersing *et al.*, 2008 ▸), memory devices (Amanuma *et al.*, 2000 ▸, Dawber *et al.*, 2005 ▸) and capacitors (Bouregba *et al.*, 2003 ▸).

The electric-field-induced properties of materials are typically determined by measuring dielectric constants (Horiuchi *et al.*, 2005 ▸) or polarization–electric field loops (Horiuchi *et al.*, 2013 ▸), whilst structural effects under applied electric fields are elucidated computationally (Li *et al.*, 2015 ▸) or using techniques such as small-angle neutron scattering (Grigoriev *et al.*, 2006 ▸). *In situ* diffraction measurements under an applied electric field provide a more complete understanding of the field-induced processes/mechanisms taking place at the structural level (Gorfman *et al.*, 2013 ▸; Usher *et al.*, 2015 ▸; Esteves *et al.*, 2015 ▸). Electric-field-induced shifts in Bragg peak position provide information about piezoelectric strain (Gorfman *et al.*, 2013 ▸; Hinterstein *et al.*, 2011 ▸). Using the whole (powder) diffraction pattern can give insight into the strain mechanism (Hinterstein *et al.*, 2015 ▸), as well as providing the possibility to investigate the response of coexisting phases (Hinterstein *et al.*, 2019 ▸). Electric-field-induced bond distortions (Gorfman *et al.*, 2013 ▸) or polarity switching (Kobayashi *et al.*, 2018 ▸) may be determined by observing the relative displacement of atoms in the crystal structure or indicated by changes in diffracted intensity (Varela *et al.*, 2000 ▸).

Several sample environments exist that allow the application of electric fields during an *in situ* diffraction experiment in house or at a central facility. These are optimized for both single-crystal and microcrystalline powders with a range of configurations. Single crystals usually have dimensions on the 1–10 mm scale and are mounted either between electrode needles (Vergentev *et al.*, 2015 ▸, 2016 ▸; Choe *et al.*, 2017 ▸) or on a sample holder with electrodes attached (Dos Santos *et al.*, 2012 ▸; van Reeuwijk *et al.*, 2000 ▸; Marchenkov *et al.*, 2018 ▸). The latter is also frequently used for powder samples, which are usually in the form of pellets. Full crystal structure determination under applied electric fields is still relatively uncommon and is less often the focus of a measurement; field-induced structural shifts or distortions tend to be very small, requiring the diffraction intensities instead to be probed.

In this contribution, we present a new sample environment on beamline I19-2, Diamond Light Source (Nowell *et al.*, 2012 ▸), which allows full structure determination from single-crystal samples under an applied electric field. Acknowledging the existing setups, here we aim to extend the *in situ* capabilities of the I19-2 beamline to electric field measurements and to better suit the small-molecule chemical crystallography user community, whose samples are typically of the order of less than 1 mm. The I19 electric field (ELF) sample cell permits the application of static/alternating fields (DC/AC) up to 4 kV with an opening angle to X-rays of *ca* 250°. We present the design elements and include a case study to show its potential for *in situ* measurements. This new sample environment makes advances in the application of electric fields to those samples on the sub-millimetre scale, whilst offering the opportunity to study processes on microsecond timescales when combined with the I19-2 time-resolved mode.

## 2. *In situ* electric field application on I19-2   

**Figure 1:**
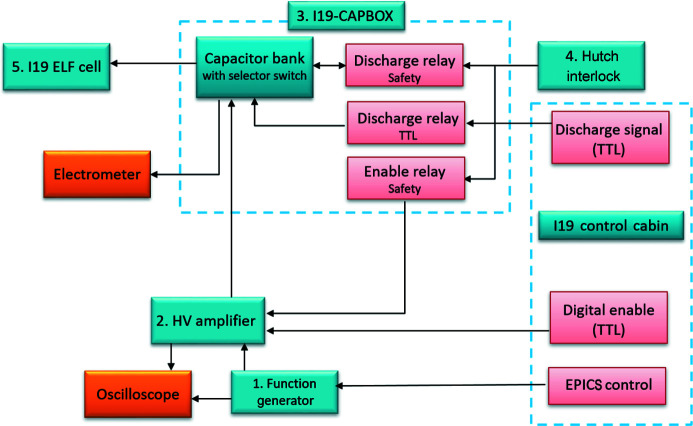
Schematic of the I19 ELF hardware on the I19-2 beamline, including (2) a high-voltage (HV) amplifier and (3) an intermediate capacitor bank (I19-CAPBOX). Connections (arrows) and control signals [TTL: transistor–transistor logic signal; *EPICS*:* Experimental Physics and Industrial Control System* (http://www.aps.anl.gov/epics/)] are also shown. Colour scheme: operating components (blue), monitoring devices (orange), signals (red).

The schematic in Fig. 1 ▸ shows the hardware configuration for applying an electric field to a sample *in situ* during an X-ray diffraction experiment on beamline I19-2 at Diamond Light Source, UK. The basic electrical connections are based on a Sawyer–Tower circuit (Sawyer & Tower, 1930 ▸). The sample is connected in series to a voltage supply (generating the electric field), with the capability to measure the sample response to electric field via a reference capacitor. The generation of the electric field starts at a function/arbitrary waveform generator (AGILENT 33210A 10 MHz). This device allows the characteristics of the electric field at the sample to be controlled and varied. The user selects the function (pulse, sinewave, ramp *etc.*), frequency (Hz) and amplitude (volts) of an initial low-voltage signal. Once programmed, this signal is output to the high-voltage amplifier (TREK model 610E) via a bayonet Neill–Concelman (BNC) double-ended cable to the amplifier’s external signal input connector (AMP INPUT Receptacle). The amplifier steps up the low-voltage signal to a 1000-fold-amplified high-voltage output.

The high-voltage output is supplied to the sample via an intermediate capacitor bank, designed in house (Fig. 1 ▸: I19-CAPBOX). The I19-CAPBOX is designed to include both safety and control features. The safety features prevent user access to the I19 ELF cell during voltage loading. This is achieved by the I19-CAPBOX forming an intermediate connection between the experiment hutch interlock, the high-voltage amplifier and the I19 ELF cell. The I19-CAPBOX receives a relay signal from the hutch interlock and only enables the high-voltage amplifier via the external control input when the hutch is in an interlocked state. The same principle is used for receiving X-rays from the synchrotron. The I19-CAPBOX also provides electrical protection to the system through a surge protector which prevents very high voltages from reaching delicate components in the circuit, such as the electrometer. It also incorporates a selection of reference capacitors in a Sawyer–Tower circuit configuration, with which polarization loops of ferroelectric materials may be recorded simultaneously (XMaS; https://warwick.ac.uk/fac/cross_fac/xmas/xmas_offline/electrical_measurements). This reference capacitor is automatically reset following a voltage-loading experiment on breaking the hutch interlock, as a further safety control (ensuring there are no charged components remaining during sample-cell exchange). The I19-CAPBOX has high-voltage output and grounding connectors to which high-voltage cabling can be connected for the attachment of the sample cell during voltage loading. In the current configuration, voltages up to 4000 V can be generated for use in an experiment.

### 2.1. I19 electric field cell   

#### 2.1.1. Sample holder   

**Figure 2:**
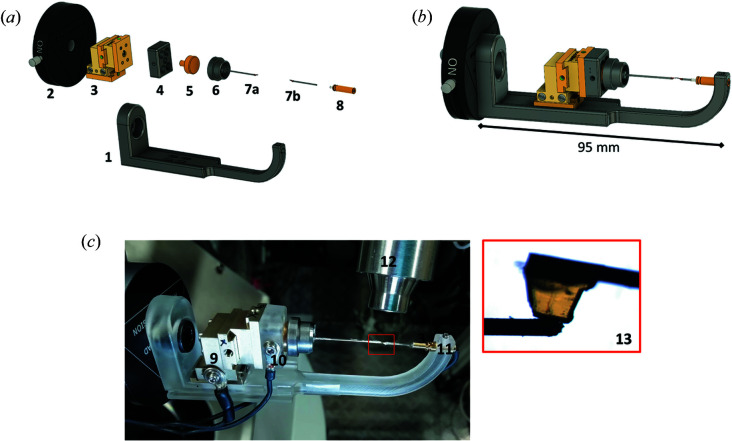
(*a*) An exploded representation of the I19 ELF cell including the following component parts: (1) 3D-printed bracket holding electrodes in position, (2) kinematic base for easy mounting, (3) Elliot Scientific/Martock MDE269 micropositioner stage for electrode–crystal alignment, (4) 3D-printed mounting block, (5) magnetic mount, (6) magnetic goniometer base with (7a) sample electrode 1 attached, (7b) electrode 2 and (8) brass electrode holder. (*b*) The assembled I19 ELF cell. (*c*) A photograph of the I19 ELF cell mounted on the I19-2 diffractometer with (9), (10) grounding and (11) high-voltage wires attached. (12) Cryostream in position for *in situ* temperature control (80–500 K). (13) An example crystal [dimensions: 0.60 (1) × 0.60 (1) × 0.20 (1) mm] mounted between electrodes.

The I19 ELF cell is based on a previous design by Vergentev *et al.* (2015 ▸) in which a single crystal is mounted between two collinear electrodes which are held in place by a mounting bracket. For the I19 ELF cell (Fig. 2 ▸), the mounting bracket is streamlined (dimensions 95 × 30 × 15 mm) to optimize the accessible region of reciprocal space (the opening angle to diffraction at kappa 0° is 250° of a φ/ω scan). This has been achieved by using 3D printing, allowing the mounting bracket design to be quickly and cheaply optimized. The bracket is 3D printed from FormLabs resin plastic, which retains a rigid structure to maintain sample centring. As the cell bracket passes through the X-ray beam it causes some shading of the diffraction images (see Section 2.3). This shading is low owing to the use of the resin plastic material and is kept consistent across measurements by mounting the sample cell on the diffractometer always in the same orientation and using a level bar to maintain the same position within tolerances of human error. This mounting method also ensures that the cell is in its expected position for the start of the data collection to provide safe movement through the data collection run list.

#### 2.1.2. Electrical connections   

**Figure 3:**
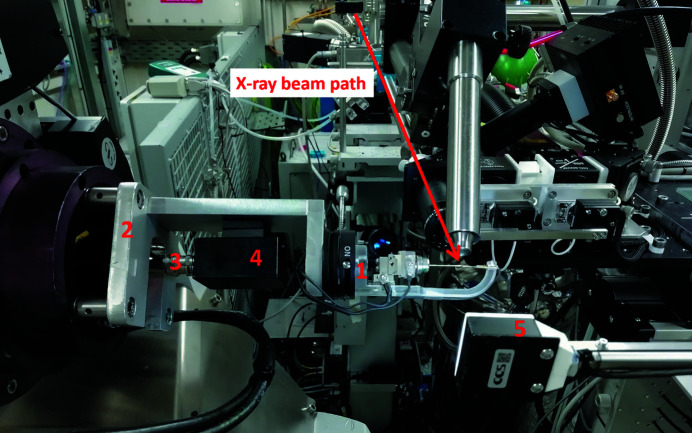
I19 ELF cell in operation. (1) The cell mounted in position on the I19-2 diffractometer, (2) the metal stand attached to the diffractometer, (3) cables from the I19-CAPBOX (fed through and secured in place in the I19-2 diffractometer), (4) the junction box, which connects the slim cell wiring with the high-voltage input and ground cables, and (5) a backlight, which moves into position for crystal illumination during sample centring. The X-ray beam path is highlighted (red arrow). The Pilatus 300K X-ray detector is not in position in the image but, during a diffraction experiment, is moved into position 5 for data collection and the backlight is moved out.

One sample electrode is detachable from the magnetic goniometer base, allowing crystal mounting offline, and sits in the cell on an Elliot Scientific/Martock MDE269 three-axis ultra-small xyz micropositioner stage to facilitate alignment and crystal docking to the second electrode, which is held in position by a brass pin. The I19 ELF cell is connected to the I19-CAPBOX *via* high-voltage cabling fed through and secured in the I19-2 diffractometer. Voltage is delivered to the sample through the use of a junction box, which forms the connection between the high-voltage cabling from the I19-CAPBOX and the slimline wiring connected to the sample electrodes on the I19 ELF cell. The sample cell with junction box attachment is mounted onto the I19-2 diffractometer on a metal stand support (Fig. 3 ▸) with kinematic magnets for ease of mounting.

#### 2.1.3. Electrode preparation   

**Figure 4:**
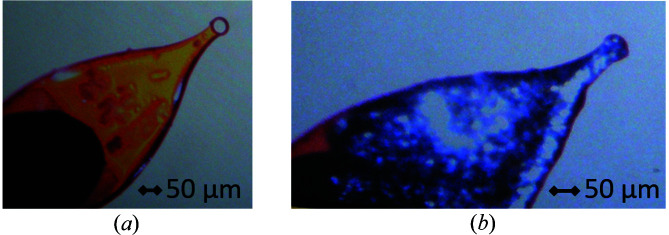
Sample pin electrode preparation before (*a*) and after (*b*) using silver paint to coat a MicroMount/Loop.

The electrodes are two industry-standard pin loops, such as the Mitegen MicroMount/Loop, which are pre-coated at the tip in conductive paint such as Electrolube Silver Conductive Adhesive paint (Fig. 4 ▸). The sample pin electrodes are glued into either the goniometer base or brass holder. Electrical connections are then ensured between the electrode and the holder by connecting lines of silver paint. This setup also allows for alternative electrodes to be used, such as graphite fibres or steel pins, which may be attached to the silver-coated loops or inserted directly into the base or brass holder, using a conductive adhesive.

#### 2.1.4. Sample preparation   

Single crystals selected for mounting in the I19 ELF cell should be manipulated dry or in the mother liquor before electrode attachment. This ensures optimum connections between the crystal and electrodes both in terms of securing in place (during gluing) and for electric field transfer. Currently the cell is optimized for crystals of at least 100 µm in all directions. There is no maximum limit in sample size, but those significantly larger than the beam size (190 × 130 µm) will cause problems with absorption effects on the diffracted intensities, introducing systematic error in their measurement. For mounting, the detachable electrode 1 tip is dipped into a mixture of silver paint and epoxy (conductive adhesive) and touched against the crystal to form the first contact. The conductive adhesive mixture is preferred for secure mounting as using silver paint alone increases the likelihood of broken contacts owing to ELF cell movement during handling. This conductive adhesive mixture is left to dry (5–10 min) before the detachable electrode is mounted in the cell. Using a microscope, the tip of electrode 2 is painted with the conductive adhesive, against which the crystal is then docked using the Elliot Scientific/Martock MDE269 micropositioner stage, adjusted using a hex key. This conductive adhesive mixture is again left to dry (5–10 min). Additional contacts between the electrode and crystal can be formed by the further addition of silver paint. One drawback of this choice of conductive adhesive is that the silver component generates powder rings in the diffraction pattern (see Section 2.3). Once the crystal–electrode contacts are dry, the I19 ELF cell is mounted onto the diffractometer using the kinematic magnet mount. Crystal centring is then performed using the in-house general data acquisition (GDA) software (Gibbons *et al.*, 2012 ▸).

#### 2.1.5. Crystal orientation   

**Figure 5:**
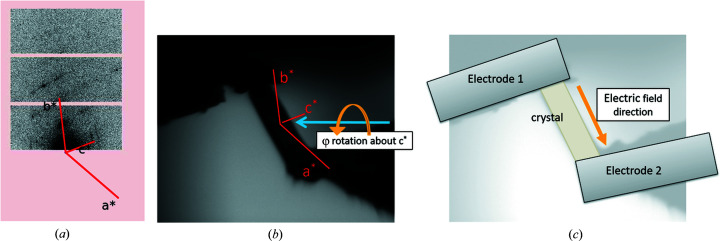
(*a*) Relationship between reciprocal lattice vectors in a diffraction image viewed in the *DIALS* image viewer, and (*b*) the corresponding crystal orientation between electrodes in the electric field cell, viewed using the on-axis viewing camera. (*c*) The equivalent schematic with electrodes, crystal and electric field direction labelled. This information can be used as a guide to indicate which crystallographic axis the electric field is being applied along and which axis, or combination of axes, electric-field-induced changes are likely to be observed in (Tazaki *et al.*, 2009 ▸).

The response of a crystalline material to an electric field is most often dependent on the orientation of the applied electric field with respect to the crystal lattice (Horiuchi & Ishibashi, 2020 ▸; Tazaki *et al.*, 2009 ▸; Owczarek *et al.*, 2016 ▸). A crystal should therefore be mounted in the cell in such an orientation that the axis of interest coincides with the direction of the applied electric field. It is recommended to perform face indexing on crystals for use in the I19 ELF cell to obtain knowledge of crystal morphology versus crystal lattice/structure orientation. This can be carried out prior to beamtime on an in-house instrument or during the beamtime on I19-2 by performing a single φ scan on a crystal perpendicular to the X-ray beam. At I19-2, this rotation scan is performed twice, once to collect diffraction images and a second time to collect on-axis camera images. Indexing is performed from the diffraction images and the indexed reciprocal lattice vectors are overlaid onto the diffraction images in the *DIALS* (*Diffraction Integration for Advanced Light Sources*; Winter *et al.*, 2018 ▸) image-viewer software. By comparing the rotation of the reciprocal lattice vectors with the corresponding crystal rotation in the camera images (Fig. 5 ▸), the crystal morphology can be compared with the crystal structure. The tool BFDH (Bravais, Friedel, Donnay and Harker) in *Mercury* (Macrae *et al.*, 2006 ▸, 2020 ▸) can also be a useful alternative, relating crystal structure to calculated morphology.

### 2.2. User controls and monitoring   

Once the I19 ELF cell is mounted on the diffractometer, the full experiment can be controlled remotely from the I19 control cabin. Control of the voltage applied to the sample is achieved by operation of the high-voltage amplifier in ‘Remote’ mode and using on/off TTL (transistor–transistor logic) signals sent via scripts incorporated into the GDA software. The reference capacitor can also be discharged on demand using a TTL signal sent via a script in the GDA software, allowing the system to be reset for further voltage loading or sample exchange. The voltage to be amplified is programmed in the arbitrary waveform function generator using an *Experimental Physics and Industrial Control System* (*EPICS*; http://www.aps.anl.gov/epics/) interface built with *Extensible Display Manager* (*EDM*; Sinclair, 2007 ▸). The programmed output from the function generator is then monitored using an oscilloscope. Oscilloscopes are further used to monitor the high-voltage amplifier outputs of the voltage (*V*_0_) and current (*I*_0_), stepped down 1000-fold. The amplifier also has a meter display on the front panel showing the amplified voltage output. The two oscilloscopes and amplifier meter panel can be monitored from the control cabin using one of the beamline webcams located inside the hutch. The hardware configuration has the capability to measure the sample response to electric field via the incorporation of an electrometer (Fig. 1 ▸) with the possibility of remote monitoring through the GDA software and the *EPICS* interface built with *EDM*. This capability is a necessity in extending the setup to time-resolved measurements and correlating structural changes with changes in the electronic response of the sample.

### 2.3. Data collection and processing   

X-ray diffraction data collection from samples in the I19 ELF cell is performed using the GDA software. Diffraction data are collected with the electric field initially off for a ‘ground state experiment’ and then with the electric field on for any electric-field-induced structural changes to be observed. The sample temperature can additionally be varied between 80 and 500 K using an Oxford Cryosystems Cryostream, which is carefully positioned so that its nitro­gen gas flow is optimally directed at the sample. At lower operating temperatures (<200 K), significant icing of the sample electrodes occurs when in the flow of the Cryostream for prolonged periods of time. This can lead to sample loss or degradation. This is unavoidable owing to the orientation of the sample electrodes relative to the flow of the liquid nitro­gen from the Cryostream nozzle. To mitigate against ice build-up, the ice can be cleared periodically by careful dislodging or by brief blocking of the Cryostream flow. An alternative contact cooling system (Mykhaylyk *et al.*, 2017 ▸) would be preferable but has not yet been incorporated into the current phase of the cell.

Data collections are run at the relatively high energy Rh edge (0.534 Å), selected to compress the diffraction pattern and to keep the number of 2θ detector positions to a minimum, whilst operating at an energy away from the Ag edge which would interact with the silver paint conductive adhesive used. At a single position of 2θ = 28° and detector distance = 100 mm, a diffraction resolution of 0.6 Å can be achieved. The I19-2 Newport four-circle diffractometer allows a data collection strategy to be performed that includes three φ scans (over a −176 to 108° range) at fixed ω (−33°) and varying κ (0, −42, 60°) and two ω scans at varying φ (−120, −5°) and fixed kappa (60°) positions.

**Figure 6:**
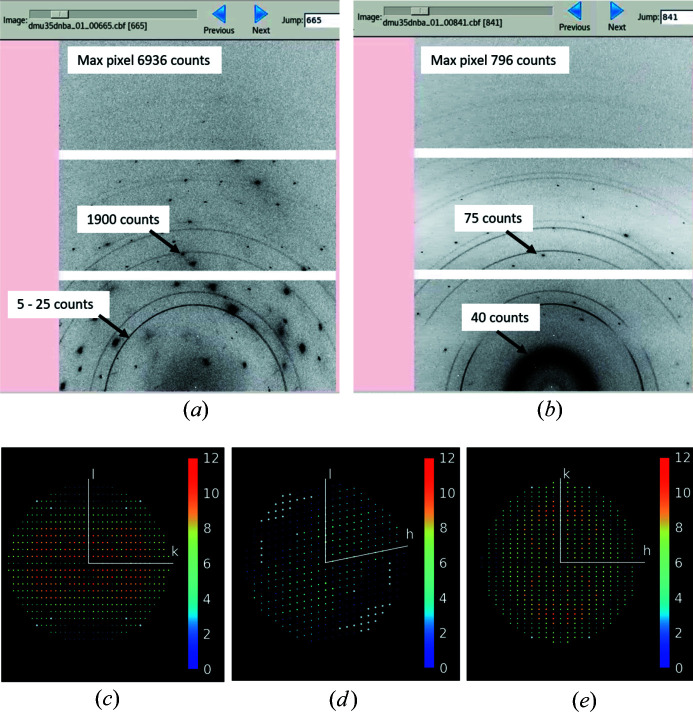
Diffraction images from a single crystal mounted in the I19 electric field cell (λ 0.534 Å and 2θ 28°). (*a*) An image free of shading from the cell and (*b*) an image shaded by the cell mounting bracket, showing how the observed diffraction is weaker. The images contain diffraction spots from the sample and powder rings from the silver component of the conductive paste. (*c*)–(*e*) *hkl* plots showing the distribution of reflection multiplicities in reciprocal space (*d* = 0.67 Å) for data collection and reduction of a data set collected from the monoclinic (*P*2_1_) system *N*,*N*-di­methyl­urea 3,5-di­nitro­benzoic acid (Saunders *et al.*, 2019 ▸) at 300 K. Reflections are coloured according to their multiplicity (0–12; see bar on the right of each image).

Because of the way that the I19 ELF cell is designed, powder rings from the silver conductive adhesive and shading from the cell bracket occur on a proportion of the diffraction images [Figs. 6 ▸(*a*) and 6 ▸(*b*)]. This leads to a reduction in diffraction intensity in the affected images. Despite this, it is possible to collect diffraction data from monoclinic or higher-symmetry systems with a good coverage of reciprocal space [Figs. 6 ▸(*c*)–6 ▸(*e*) and Fig. S1 in the supporting information]. The powder rings and shading from the I19 ELF cell in the diffraction images can be accounted for in the data processing, which is performed using *xia2* (Winter, 2010 ▸) with *DIALS* (Winter *et al.*, 2018 ▸). For weakly diffracting samples, it is recommended to use a combination of masking and the removal of sections of the shaded data during the data processing. This can result in a reduced completeness of the diffraction data but improved merging statistics. For strongly diffracting samples with a low mosaic spread, good data processing statistics can be achieved using the default *xia2*/*DIALS* settings on all of the diffraction data (Table 1 ▸). *DIALS* treats the affected data initially during the spot finding, where affected intensities are either undetected or rejected on the basis of a maximum peak-to-centroid separation (in pixels) criterion. Later, in the *DIALS* scaling routine, any affected intensities are subject to further rejection if they deviate significantly from the expected Wilson distribution (Wilson, 1942 ▸; Giacovazzo *et al.*, 2011 ▸).

## 3. Case study: electric-field-induced colour change in single crystals of 4,4′-bipyridinium hydrogen squarate   

**Figure 7:**
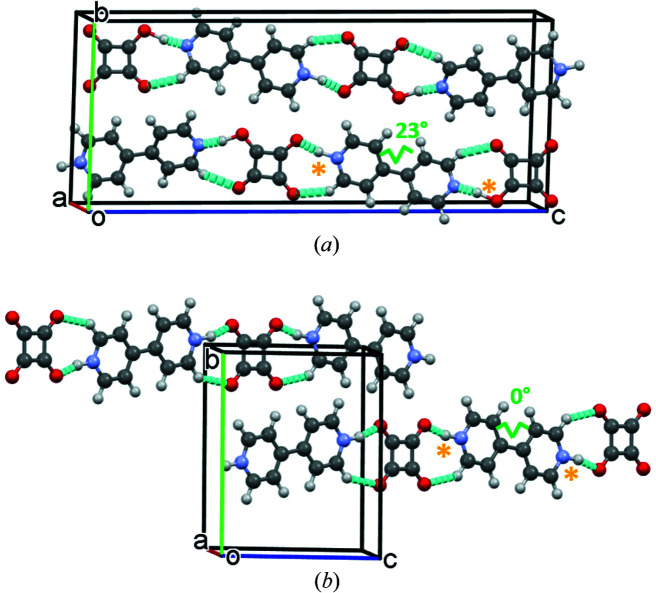
Hydrogen-bonded chains in the crystal structures of (*a*) SQABPY-I [*P*2_1_/*n* form; Cambridge Structural Database (CSD) refcode HAZFAP01; Martins *et al.*, 2009 ▸] and (*b*) SQABPY-II (*C*2/*c* form; CSD refcode HAZFAP07; Martins *et al.*, 2009 ▸), showing the differing protonation states (orange asterisks) and molecular torsions of bi­pyridine rings (green lines). Hydrogen bonds are drawn as dashed cyan lines.

The crystallization of squaric acid with 4,4′-bi­pyridine generates a 1:1 adduct where, at room temperature, the acid and bi­pyridine molecules are present in their monoprotonated forms in space group *P*2_1_/*n* (SQABPY-I). Single crystals of SQABPY-I are in the form of rectangular needles and are yellow ochre in colour (Reetz *et al.*, 1994 ▸). This system has previously been shown to exhibit temperature- and pressure-induced proton-transfer behaviour by single-crystal X-ray diffraction, neutron powder diffraction, and optical and infrared spectroscopy (Martins *et al.*, 2009 ▸). The proton-transfer event occurs along hydrogen-bonded chains in the crystal structure from the monoprotonated squarate to the monoprotonated bipyridinium to form a diprotonated bipyridinium ion (Fig. 7 ▸) and is reversible. The proton transfer is coupled with a crystallographic phase transition from space group *P*2_1_/*n* (SQABPY-I) to *C*2/*c* (SQABPY-II) and significant changes in lattice parameters [from *P*2_1_/*n*, *a* = 3.8000 (10), *b* = 11.2080 (10), *c* = 27.447 (2) Å, β = 92.220 (10)° to *C*2/*c*, * a* = 12.465 (25), *b* = 11.2747 (11), *c* = 9.0706 (20) Å, β = 109.497 (13)°]. An associated colour change occurs during the phase transition, where the yellow SQABPY-I crystals turn to red in the SQABPY-II phase. This is thought to be caused by a narrowing of the squarate–bipyridinium charge-transfer energy gap following the proton transfer. The powder X-ray diffraction and differential scanning calorimetry measurements conducted by Martins *et al.* (2009 ▸), as well as confirming the reversibility of the phase transition, suggest a significant kinetic barrier for the conversion from SQABPY-II back to SQABPY-I; a hysteresis occurs on cooling below the phase-transition temperature, whilst subsequent cooling–heating cycles reveal a reduction in the energy change for the transition from 5.4 to 4.2 kJ mol^−1^.

The susceptibility of SQABPY-I to external stimuli makes it a good candidate for electric field studies in the I19 ELF cell. Proton-transfer behaviour is also known to occur under applied electric fields in ferroelectric materials, in which proton shuttling may facilitate the reversal of material polarity (Horiuchi *et al.*, 2010 ▸, 2017 ▸; Abronin *et al.*, 2016 ▸) or lead to transitions between electric states, including paraferro– (Yao *et al.*, 2016 ▸; Horiuchi *et al.*, 2005 ▸) or antiferro–ferroelectric (Horiuchi, Tsutsumi*, et al.*, 2018 ▸). Extended hydrogen-bonded chains of acid–base molecules (such as formed in SQABPY-I) can further favour proton shuttling under an applied electric field (Horiuchi *et al.*, 2009 ▸; Horiuchi & Ishibashi, 2020 ▸), whilst squaric acid is found in single-component (Horiuchi, Kumai & Ishibashi, 2018 ▸) and multi-component proton-transfer materials with multiple electric states (Lengyel *et al.*, 2019 ▸). Materials such as these offer interesting applications, including in optical communications (Miyamoto *et al.*, 2018 ▸) and high-power energy-storage systems (Horiuchi, Kumai & Ishibashi, 2018 ▸) and for electrostriction applications (Kobayashi *et al.*, 2018 ▸).

### 3.1. Experimental   

Single crystals of SQABPY-I were prepared by dissolving equimolar quantities of squaric acid and 4,4′-bi­pyridine in H_2_O heated to 60°C and stirring continuously. Initially, a bright-orange precipitate formed as the squaric acid immediately (singly) protonates the bi­pyridine. This precipitate dissolved after approximately one hour of continuous heating and stirring (combining 2.5 mmol of each component gives a reacted product that dissolves in ∼50 ml of H_2_O at 60°C). Slow cooling of the solution and evaporation (approximately two months) produces a number of large rectangular planks. A large volume of small needles can be grown by crash cooling (minutes to hours) of a concentrated hot aqueous solution (2.5 mmol of each component in 20 ml of H_2_O at 90°C).

Single-crystal X-ray diffraction data were collected on crystals of SQABPY-I in the I19 ELF cell on beamline I19-2 at Diamond Light Source, UK, using a Newport four-circle diffractometer equipped with a PILATUS 300K detector and an energy of λ = 0.534 Å. Diffraction data were measured from the sample at room temperature. Data collection was performed using the in-house GDA software, and data were processed using *xia2* for small molecules with additional *DIALS* commands to input the unit cell and space group and a resolution cut-off of 0.75 Å (see Table S3). Structures were solved using *SHELXT* (Sheldrick, 2015*a* ▸) and refined using *SHELXL* (Sheldrick, 2015*b* ▸) in *OLEX2-1.3* (Dolomanov *et al.*, 2009 ▸). H-atom refinement details are included in the supporting information (Table S4).

Face indexing of the crystals was carried out using a Rigaku Oxford Diffraction (formerly Agilent Technologies) Supernova diffractometer with Mo *K*α (0.71073 Å) radiation, equipped with an optical camera to select the crystal faces. The *CrysAlisPro* (1.171.40.84a; Rigaku Oxford Diffraction) software was used to index the crystal faces. *Mercury* was used to calculate the BFDH morphology and to determine the mol­ecular arrangement relative to the crystal faces and unit-cell axes.

### 3.2. Crystal habit   

Crystals of SQABPY-I grow as rectangular small needles or large planks (depending on the crystallization method) and always with a long length, a narrow edge and a dominant large face, corresponding to crystal width. To relate crystal structure to crystal habit, face indexing was performed on several crystals of SQABPY-I.

**Figure 8:**
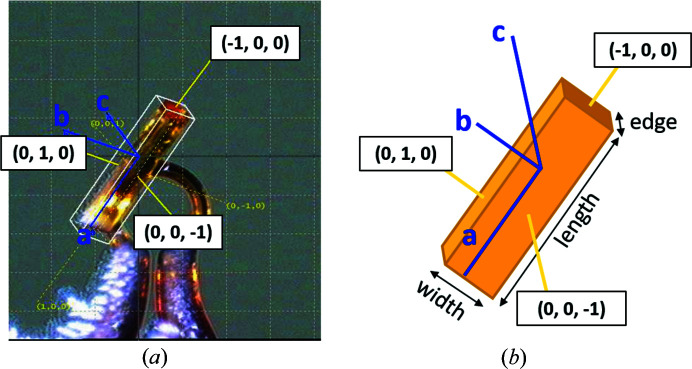
A face-indexed crystal of SQABPY-I: (*a*) a small needle grown by fast cooling and (*b*) a pictogram depiction. Miller indices (yellow lines) of each crystal face (defined by the white box) alongside the orientation of unit-cell axes (blue lines) relative to crystal faces are shown. Face indexing was performed within the *CrysAlisPro* software (1.171.40.84a) using the face-indexing tool.

The faces of the SQABPY-I needles are identified as (100), (010) and (001) in all measured samples (Fig. 8 ▸ and Fig. S2). The long needle length corresponds to the crystallographic *a* axis, capped by the (100) and (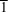
00) faces. The narrow crystal edges correspond to the (010) and (0
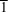
0) faces and are perpendicular to the crystallographic *b* axis. The dominant crystal width corresponds to the (001) and (00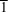) faces which are perpendicular to the crystallographic *c* axis. The BFDH *Mercury* crystal morphology correctly predicts the *a* axis to be the longest length of the crystal and the *c* axis to be perpendicular to the crystal width (Fig. S3). This tool therefore has potential for the correct assignment of unit-cell orientation relative to crystal faces where an extreme axis is present.

### 3.3. Offline electric field application   

Offline optical measurements were first made to test the response of the SQABPY-I crystals to the electric field and to determine if a voltage-induced colour change could be observed. A single crystal [dimensions: 0.60 (1) × 0.60 (1) × 0.20 (1) mm] cut from a needle of SQABPY-I was mounted in the I19 ELF cell following the procedure outlined in Section 2.1. The SQABPY-I crystal habit favoured their mounting in the I19 ELF cell with electrodes attached to the (010) and (0
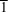
0) faces such that the electric field was applied parallel to the crystallographic *b* axis.

**Figure 9:**
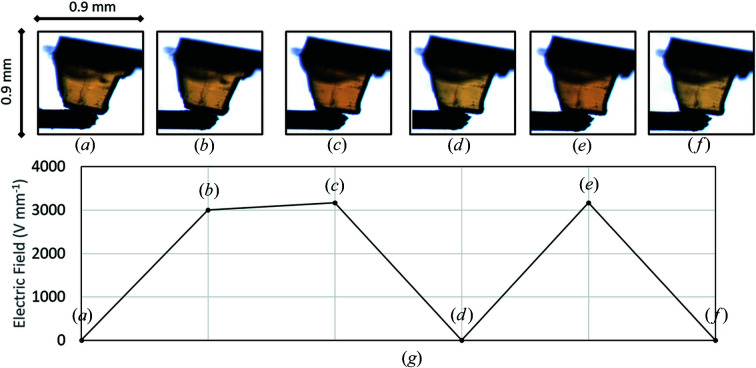
A single-crystal sample of SQABPY-I (cut from a larger plank) mounted in the I19 ELF cell during voltage ramping at room temperature (the sample corresponds to crystal 02, Table 2 ▸). The crystal remained yellow up to 1800 V (*a*), (*b*). At 1900 V (*c*), a yellow to red-shifted yellow colour change occurs, which is reversible and repeatable with further voltage off/on (*d*)–(*f*). (*g*) Plot of electric field (V mm^−1^) versus crystal appearance.

Once the crystal had been mounted, voltage ramping was performed at room temperature and the crystal was monitored for changes using the on-axis viewing camera (Fig. 9 ▸). The voltage was increased stepwise (200 V steps) from 0 to 1800 V. The crystal remained in its yellow form up to 1800 V. At 1900 V (≃ 3000 V mm^−1^) [Fig. 9 ▸(*c*)], the crystal appeared to shorten parallel to the direction of field application, with an accompanying subtle colour change from yellow to a red-shifted yellow. This colour change was reversible as the voltage supply to the crystal was turned off and on [Figs. 9 ▸(*d*)–9 ▸(*f*)]. No further colour change was observed on increasing the voltage to 2100 V (3500 V mm^−1^), where the electric field began to break down. Average colour picker analysis (https://matkl.github.io/average-color/) from an area of the crystal in images (*a*), (*c*) and (*d*) in Fig. 9 ▸ identifies a difference in colour with voltage application (Fig. S4). Initial attempts have been made to quantify the colour change using UV–Vis spectroscopy; however, this setup is still in the early commissioning phases and so no conclusions can yet be drawn from the measurements.

A number of SQABPY-I crystals of different sizes were tested offline for this colour-change behaviour. It was found that the field gap (corresponding to crystal width) affected the point at which the colour change occurred; the larger the crystal, the greater the voltage required to switch the sample (Table 2 ▸). The critical field of switching might be expected to remain constant. However, this is not the case here and can be attributed to differences in sample alignment between electrodes or variations in ‘actual’ voltage being felt by the crystal (there may be slight variations in conductivity between sample cells).

### 3.4. *In situ* diffraction measurements   

To characterize the electric-field-induced colour change in SQABPY-I, *in situ* single-crystal X-ray diffraction measurements were performed on beamline I19-2, Diamond Light Source, at room temperature on a single crystal [crystal 03 in Table 3 ▸; dimensions 2.50 (1) × 1.00 (1) × 0.10 (1) µm] of SQABPY-I mounted in the I19 ELF cell. The SQABPY-I crystal was mounted in the cell in the known field-responsive orientation, *i.e.* such that the electrodes were attached to the (010) and (0
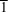
0) faces and field application was along the crystallographic *b*-axis direction (see Section 3.2). The diffractometer rotation axis coincided with the crystallographic *a* axis.

**Figure 10:**
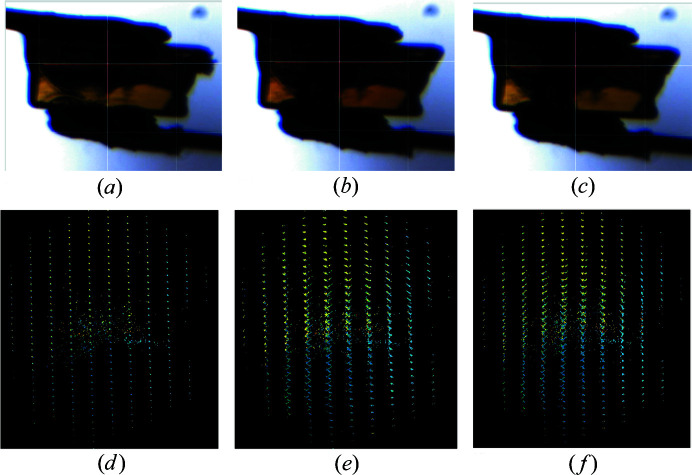
A single-crystal sample of SQABPY-I measured *in situ* in the I19 ELF cell during voltage ramping and data collection on beamline I19-2, Diamond Light Source. (*a*) Before-voltage (0 V) yellow form, (*b*) during-high-voltage (2400 V) red-shifted yellow form and (*c*) after-voltage-off (0 V) yellow form. The reciprocal lattice of diffraction spots (as viewed down the *c* axis in the *DIALS* reciprocal lattice viewer) from the data processing spot-finding routine for (*d*) the before-voltage (0 V) yellow form, (*e*) the during-high-voltage (2400 V) red-shifted yellow form and (*f*) the after-voltage (0 V) yellow form.

Diffraction data were collected before, during and after the application of electric field. Initially, the crystal was yellow, as expected for SQABPY-I. Upon application of 2400 V, a red shift in the colour was observed, which returned to yellow when the voltage was switched off [Figs. 10 ▸(*a*)–10 ▸(*c*)]. The diffraction data indicated some irreversible change in mosaic spread of the crystal by the twinning of diffraction spots [Figs. 10 ▸(*d*)–10 ▸(*f*)] and a reduction in the data quality, in particular a significant increase in *R*_merge_ suggesting a worse agreement between equivalent reflections (Table 3 ▸), during and after the application of the electric field.

**Figure 11:**
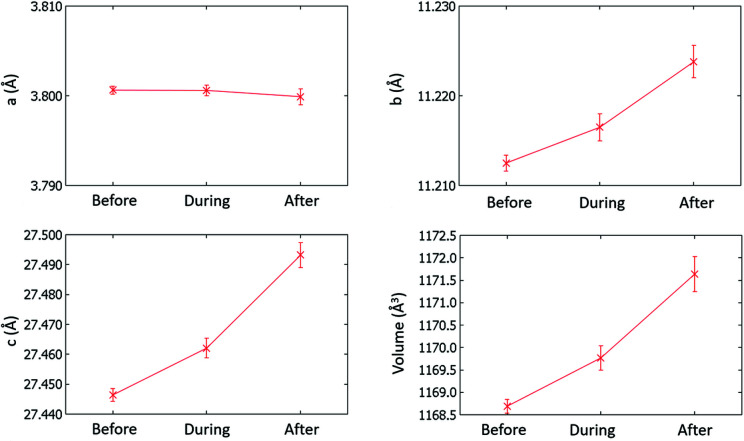
Unit-cell parameters and volume before (yellow form), during (high-voltage red-shifted yellow form) and after (yellow form) applying an electric field of 2400 V mm^−1^. Error bars represent three standard deviations (3σ).

The consistent unit-cell parameters and space group for the before-, during- and post-voltage forms indicate that there are no large structural changes occurring as a function of voltage (Fig. 11 ▸ and Table S5). The most significant change occurs in the *c* axis, which, between before voltage and during voltage on, lengthens by 0.02 Å (a change of 0.07%). After the voltage is turned off, the unit-cell parameters do not relax to their start values. This may be a factor of the irreversible twinning of the crystal post voltage application. However, it could also be caused by remnant voltage effects felt by the crystal as the ‘after-voltage’ data collection was performed immediately after turning the voltage off with a maximum delay of minutes, the time taken for the diffractometer to move to the data collection start position. A longer delay may have allowed the crystal to relax to its initial state (Lau *et al.*, 2015 ▸), though this can be between hours and days and was beyond the allowed time of the experiment.

The *c* axis coincides most with the direction of the hydrogen-bonded chain (Fig. 7 ▸). This prompted a closer look at the atomic coordinates of SQABPY-1, to examine if any structural changes had occurred and if they bore any similarity to those observed in the thermal phase transition between SQABPY-I and SQABPY-II.

**Figure 12:**
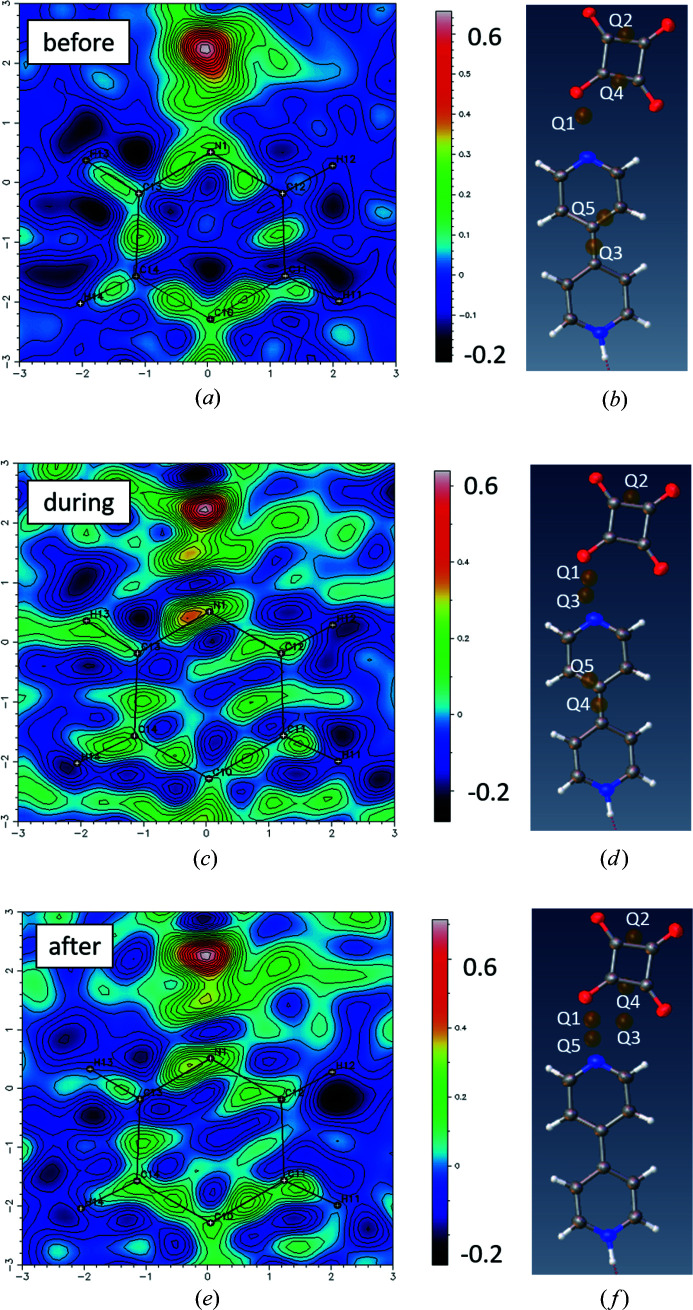
Residual electron density maps generated in the plane of the pyridinium C—N—C atoms and in the region of the O—H⋯N hydrogen bond formed between the hydrogen squarate and the un-protonated 4,4′-bipyridinium nitro­gen atom for (*a*), (*b*) the before-voltage (0 V) yellow form, (*c*), (*d*) the during-high-voltage (2400 V) red-shifted yellow form and (*e*), (*f*) the after-voltage-off (0 V) yellow form. Residuals are indicated as maxima (red regions) in the Fourier difference electron density maps (*a*), (*c*), (*e*) or as *Q* peaks (brown spheres) visualized in *Olex2-1.3* (*b*), (*d*), (*f*). The O—H⋯N H atom is omitted from the model.

The crystal structures for the before, during and after forms show that there is no shift in the non-H-atom positions as a function of voltage (Fig. S5). There is, however, residual electron density located in the bonding region of the un-protonated 4,4′-bipyridinium nitro­gen atom (Fig. 12 ▸) in the during- and after-voltage structures, apparent when the hydrogen squarate proton is left un-modelled. This residual electron density is evident in both Fourier difference maps and is indicated by a *Q* peak following *SHELXL* refinement in *Olex2-1.3*. This peak of residual electron density indicates a potential disorder of this proton across the O—H⋯N hydrogen bond to the un-protonated 4,4′-bipyridinium nitro­gen atom. The second hydrogen atom peak has the greatest intensity for the during-high-voltage form, suggesting that it is caused by the application of the electric field. The fact that it remains to an extent in the subsequent after-voltage-off form indicates that the crystal has not yet fully ‘relaxed’ after the voltage being turned off (as seen in the unit-cell parameters).

To check the likelihood of electric-field-induced proton disorder, a proton disorder model was refined for all three forms (see Table S4 for the H-atom model used). A stable disorder model was only achieved for the during-high-voltage form; the second hydrogen atom occupied a chemically sensible position, in the plane of the bi­pyridine ring. The occupancies of the major (on the acid) and minor (on the bi­pyridine) disordered proton sites refined to a 80:20 split, indicating a low but present occupation of the second H-atom site in the O—H⋯N hydrogen bond as a result of the applied electric field. In contrast, when applying the same disorder model to the before- and after-voltage forms, the H atom deviates from being in a chemically sensible position, lifting up and out of the plane of the bi­pyridine ring it is bonded to. An unstable model suggests that proton disorder is most likely absent in the before- and after-high-voltage forms.

Indexing of the crystal habit shows that the electric field was applied parallel to the crystallographic *b* axis, perpendicular to the (010) and (0
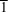
0) faces. This axis is almost perpendicular to the hydrogen-bonding direction (Fig. 7 ▸) and may explain why only a small extent of proton disorder is observed following electric field application. Future measurements targeting crystal alignment such that the electrodes are attached to the (001) and (00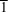) faces and the electric field is applied parallel to the crystallographic *c* axis could result in a greater disorder of the protons, possibly to the extent that the red SQABPY-II form is accessible. This will be the focus of follow-up studies on this system.

**Figure 13:**
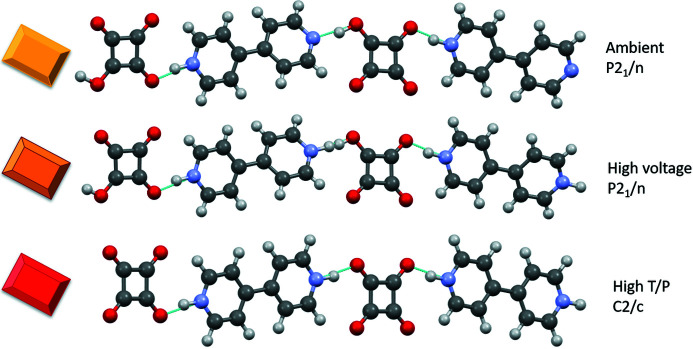
Summary of structures of squarate 4,4′-bipyridinium salts including a yellow *P*2_1_/*n* form (CSD refcode HAZFAP01; Martins *et al.*, 2009 ▸) at ambient conditions, a high-voltage red-shifted yellow *P*2_1_/*n* form and a high-temperature/pressure red *C*2/*c* form (CSD refcode HAZFAP07; Martins *et al.*, 2009 ▸).

The early evidence presented here suggests that a small extent of proton disorder may be responsible for the colour change observed on application of an electric field to SQABPY-I. As determined by the *in situ* diffraction measurements; the field leads to a proton hopping of the second hydrogen squarate proton towards the monoprotonated 4,4′-bipyridinium molecule. In the extreme, full hopping would result in a structure containing both the squarate anion and the doubly protonated 4,4′-bipyridinium molecule, similar to the high-temperature red form, SQABPY-II. It is therefore reasonable that a partially proton transferred state could lead to the intermediate red-shifted yellow form (Fig. 13 ▸) which is achieved here at a field strength of 2.4 kV mm^−1^, although it should be noted that the critical field needed to induce a visible colour change in other crystals could be higher.

## 4. Conclusions   

In this work we have presented for the first time the I19 ELF cell for use in single-crystal synchrotron X-ray diffraction measurements. We have shown how it allows the user to elucidate electric-field-induced structural responses *in situ* during a diffraction experiment. This is a significant step in electric field studies where few experimental setups exist that allow *in situ* structure determination from single crystals.

Using the I19 ELF cell, we have identified an interesting electric-field-sensitive material, SQABPY-I, found to change colour on application of an electric field. In these preliminary results, by performing *in situ* diffraction measurements, the voltage-induced colour change can be linked to the extent of proton disorder within the system. Whilst proton transfer has previously been linked to colour change in other single-crystal systems (Jones *et al.*, 2014 ▸; Yano *et al.*, 2019 ▸), cases of proton hopping as a function of electric field in organic molecular systems remain rare (Varela *et al.*, 2000 ▸; Horiuchi *et al.*, 2010 ▸). Even more so are experimental studies of proton transfer performed *in situ* during a single-crystal diffraction experiment. We continue to work on the development of the cell, for its optimization towards smaller samples and time-resolved measurements, in order to cater better to the varied I19-2 user community.

## Supplementary Material

Crystal structure: contains datablock(s) SQABPY_before_voltage_0V, SQABPY_during_high_voltage_2400V, SQABPY_after_voltage. DOI: 10.1107/S1600576721007469/oc5009sup1.cif

Structure factors: contains datablock(s) SQABPY_before_voltage_0V. DOI: 10.1107/S1600576721007469/oc5009SQABPY_before_voltage_0Vsup2.hkl

Structure factors: contains datablock(s) SQABPY_during_high_voltage_2400V. DOI: 10.1107/S1600576721007469/oc5009SQABPY_during_high_voltage_2400Vsup3.hkl

Structure factors: contains datablock(s) SQABPY_after_voltage. DOI: 10.1107/S1600576721007469/oc5009SQABPY_after_voltagesup4.hkl

Supporting information file. DOI: 10.1107/S1600576721007469/oc5009sup5.pdf

CCDC references: 2073116, 2073117, 2073118

## Figures and Tables

**Table 1:** Statistics following data reduction in *xia2* of an I19 ELF cell data set collected from the monoclinic (*P*2_1_) system *N*,*N*-di­methyl­urea 3,5-di­nitro­benzoic acid (Saunders *et al.*, 2019 ▸) at 300 K Additional data processing commands used include a resolution cut-off of 0.67 Å; full processing details are included in Tables S1 and S2 in the supporting information.

	Overall	Low resolution	High resolution
Resolution (Å)	11.48–0.67	11.48–1.81	0.68–0.67
Observations	11 755	887	415
Unique reflections	2424	139	117
Multiplicity	4.8	6.4	3.5
Completeness (%)	97.51	100.00	94.35
Mean *I*/σ(*I*)	25.8	197.4	1.0
*R*_merge_	0.025	0.009	0.360

**Table 2:** Crystal size versus voltage at which yellow to red-shifted yellow colour change is induced (switching voltage) and relative critical field

Crystal	Crystal size (mm)	Field gap (mm)	Switching voltage (V)	Critical field (V mm^−1^)
01	1.00 (1) × 0.20 (1) × 0.10 (1)	0.2	1400	7000
02	0.60 (1) × 0.60 (1) × 0.20 (1)	0.6	1900	3167
03	2.50 (1) × 1.00 (1) × 0.10 (1)	1.0	2400	2400

**Table 3:** Crystal data for the before-voltage (0 V) yellow form, the during-high-voltage (2400 V) red-shifted yellow form and the after-voltage (0 V) yellow form

	Before voltage	During high voltage	After voltage
Voltage (V)	0	2400	0
Crystal colour	Yellow	Red-shifted yellow	Yellow
Temperature (K)	298	298	298
Resolution cut-off (Å)	0.75	0.75	0.75
Crystal system	Monoclinic	Monoclinic	Monoclinic
Space group	*P*2_1_/*n*	*P*2_1_/*n*	*P*2_1_/*n*
*a* (Å)	3.80060 (10)	3.8006 (2)	3.7999 (3)
*b* (Å)	11.2125 (3)	11.2165 (5)	11.2238 (6)
*c* (Å)	27.4464 (7)	27.4621 (11)	27.4932 (14)
α (°)	90	90	90
β (°)	92.272 (3)	92.271 (5)	92.277 (6)
γ (°)	90	90	90
Volume (Å^3^)	1168.69 (5)	1169.77 (9)	1171.64 (13)
*Z*	4	4	4
ρ_calc_ (g cm^−3^)	1.536	1.534	1.532
μ (mm^−1^)	0.065	0.065	0.065
*F*(000)	560	560	560
Crystal size (mm)	2.5 × 1.0 ×0.1	2.5 × 1.0 × 0.1	2.5 × 1.0 × 0.1
Diffraction wavelength (Å)	0.534	0.534	0.534
2θ range for data collection (°)	2.232–41.586	2.23–41.788	2.228–41.82
Index ranges	−5 ≤* h *≤ 4	−5 ≤ *h* ≤ 4	−4 ≤ *h* ≤ 5
	−14 ≤ *k* ≤ 14	−14 ≤ *k* ≤ 14	−14 ≤ *k* ≤ 14
	−36 ≤ *l* ≤ 36	−36 ≤ *l* ≤ 36	−36 ≤ *l* ≤ 36
Reflections collected	15 341	15 159	15 087
Independent reflections	2791	2813	2820
	*R*_int_ = 0.0649	*R*_int_ = 0.0839	*R*_int_ = 0.0910
	*R*_sigma_ = 0.0435	*R*_sigma_ = 0.0847	*R*_sigma_ = 0.0914
Data/restraints/parameters	2791/0/222	2813/2/226	2820/0/222
Goodness-of-fit on *F* ^2^	1.129	0.828	0.822
Final *R* indices [*I* ≥ 2σ(*I*)]	*R*_1_ = 0.0418	*R*_1_ = 0.0495	*R*_1_ = 0.0493
	*wR*_2_ = 0.1096	*wR*_2_ = 0.1037	*wR*_2_ = 0.1025
Final *R* indices (all data)	*R*_1_ = 0.0526	*R*_1_ = 0.0770	*R*_1_ = 0.0758
	*wR*_2_ = 0.1203	*wR*_2_ = 0.1136	*wR*_2_ = 0.1124
Largest peak/hole difference (e Å^−3^)	0.335/−0.209	0.27/−0.22	0.306/−0.223
