# Supplementary material for: An electric field cell for performing in situ single-crystal synchrotron X-ray diffraction
Source: J Appl Crystallogr. 2021 Sep 4;54(Pt 5):1349–59. doi: 10.1107/S1600576721007469 (PMC8493620; doi:10.1107/S1600576721007469)
Supplement: Supplementary file 5 [file j-54-01349-sup5.pdf]

JOURNAL OF APPLIED CRYSTALLOGRAPHY

Volume 54 (2021)

**Supporting information for article:**

## An electric field cell for performing *in situ* single-crystal synchrotron X-ray diffraction

**Lucy K. Saunders, Hamish H. -M. Yeung, Mark R. Warren, Peter Smith, Stuart Gurney, Stephen F. Dodsworth, Inigo J. Vitorica-Yrezabal, Adrian Wilcox, Paul V. Hathaway, Geoff Preece, Paul Roberts, Sarah A. Barnett and David R. Allan**

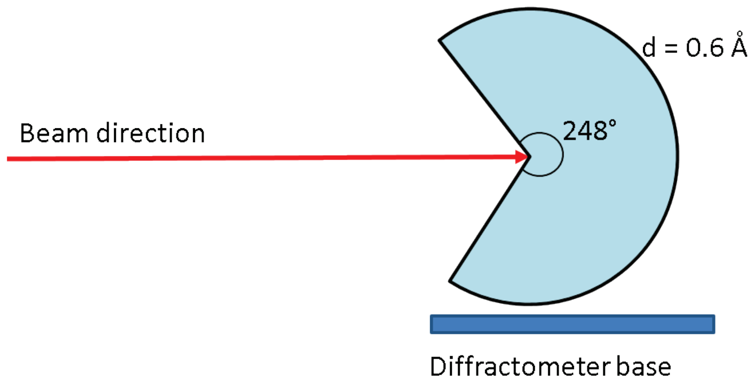


**Figure S1** The region of reciprocal space accessible during a diffraction experiment using the I19 ELF cell with the cryostream in position and a diffraction wavelength of 0.534 Å. The accessible region is a sphere with a wedge is missing as a result of the limitation of movement imposed by the cryostream and is further limited by size of detector face (achieving resolution of 0.6 Å at 2θ = 28°).

**Table S1** xia2 (Winter, 2010) for small molecule using DIALs (Winter *et al.*, 2018) data processing commands run to output example diffraction data reduction in Table 1 of the manuscript.

| Data processing commands |
| --- |
| Command line: xia2 /dls/i19-2/data/2018/cm19670-5/20181210/07_dmu35dnba02 small_molecule=True d_min=0.67 |

**Table S2** xia2 (Winter, 2010) for small molecule with DIALs (Winter *et al.*, 2018) data processing output.

Xia2 for small molecule xia2.txt file contents

Environment configuration...

Python => /dls_sw/apps/dials/dials-v3-2-0/build/../conda_base/bin/python3.8

CCTBX => /dls_sw/apps/dials/dials-v3-2-0/modules

CCP4 => /dls_sw/apps/ccp4/7.1.006/ccp4-7.1

CCP4_SCR => /tmp/1196636.1.all.q/tmpbrryptb3

Starting directory: /dls/i19-2/data/2018/cm19670-5/processing/xia2GUI/07_dmu35dnba02_20201112_1136

Working directory: /dls/i19-2/data/2018/cm19670-5/processing/xia2GUI/07_dmu35dnba02_20201112_1136

Free space: 8529030.53 GB

Host: cs05r-sc-com02-05

Contact: xia2.support@gmail.com

XIA2 0.7.102-gc8f0158d-dials-3.2

DIALS 3.2.0-g03ffb6a06

CCP4 7.1.006

Command line: xia2 /dls/i19-2/data/2018/cm19670-5/20181210/07_dmu35dnba02 small_molecule=True d_min=0.67

Project directory: /dls/i19-2/data/2018/cm19670-5/processing/xia2GUI/07_dmu35dnba02_20201112_1136

-------------------- Spotfinding SWEEP1 --------------------

6884 spots found on 1240 images (max 242 / bin)

```
       *
      ***
     ****                 *
     *****         **    *******     *
     *******        **    ********* *********
     *******       ***    *******************
    **********      *****    *******************
 *  *** ****************** *  ******* *  *******************
************************************************************
************************************************************
1                 image                 1240
```

-------------------- Spotfinding SWEEP2 --------------------

4711 spots found on 1090 images (max 234 / bin)

```
     ***
     ***
   *  *****
 * **********
 **************
***************
****************
********************     *** *   *
*********************** * *********   *  *** ****** *******
************************************************************
1                 image                 1090
```

WARNING: Potential blank images: 641 -> 650

WARNING: Potential blank images: 661 -> 710

WARNING: Potential blank images: 731 -> 750

-------------------- Spotfinding SWEEP3 --------------------

```
8186 spots found on 1030 images (max 338 / bin)
                              *
               *            ***
               ***     *  *     ***
            ***  ****    ** **    *****
            **** *****    ******    *****
           ************   ********  *******
          *************  **  ********  ********
          *************** ** **********  ********
          ***************************************
************************************************************
1             image           1030
WARNING: Potential blank images: 21 -> 40
WARNING: Potential blank images: 61 -> 100
WARNING: Potential blank images: 111 -> 210
WARNING: Potential blank images: 221 -> 270
WARNING: Potential blank images: 281 -> 290
-------------------- Spotfinding SWEEP4 --------------------
5552 spots found on 640 images (max 251 / bin)
                        *
                       ***   *
                       ***   *** ***
                       ***   *******
      **                ****  ********
   * *****              ** *****  *********
  **********              ** ****************
****************            ** ****************
****************** *        *********************
************************************************************
1             image            640
WARNING: Potential blank images: 251 -> 260
WARNING: Potential blank images: 271 -> 280
WARNING: Potential blank images: 291 -> 300
WARNING: Potential blank images: 311 -> 320
WARNING: Potential blank images: 341 -> 380
-------------------- Spotfinding SWEEP5 --------------------
10112 spots found on 640 images (max 313 / bin)
**
**   *
********** *               **
*************  **            ***
******************   *   *     ******   *  *
******************   **  ***    ******** ********
******************   **  *****   **********************
******************  *************  **********************
************************************ **********************
************************************************************
1             image            640
---------------- Autoindexing SWEEPS 1 to 5 ----------------
All possible indexing solutions:
```

mP 7.57 11.52 8.03 90.00 101.51 90.00

aP 7.57 8.03 11.53 90.03 89.93 78.50

Indexing solution:

mP 7.57 11.52 8.03 90.00 101.51 90.00

-------------------- Integrating SWEEP1 --------------------

Processed batches 2 to 1241

Standard Deviation in pixel range: 0.01 0.69

Integration status per image (60/record):

ooooooooooooooo.oooooooooooooooooo.ooooooooooooooooooooooooo

ooooooooooooooooooooooooooooooooooooooooooooooooooo.oooooooo

ooooooooooooooooooooooooo.o.oo.oooooooooooooooooooo.oooooooo

ooooooooo.ooooooooooooooooooooooooooo..o.ooooooooooooo.ooooo

ooooooooooooooo.oo.ooooooooo.ooooooooo.ooo.ooooooooooooo.oo.

ooo.ooo.oooooooooooo.ooo.ooooooooooooooooooooooooo.ooooooo.oo

oooooo.ooooooooooo.oooooooooooooooooo.o.o.ooooooo.o.oooooooo

oo.ooo.oooooooooooo.oo.oooooo.oooo.ooooo.ooo.ooooooooooooooo

oooooooooooooo.oooooooooooooooo.ooooooooooooooooooo.oooooooo

ooooooo..oooooooo.oooooooooooooooo.o.oooo.oooooooo.oo.oooooo

oooooooo.oo.ooooooooooooooooooooooooo.ooooooooooo.oo.ooooooo

oooo.ooooooooooooooo.ooooooooo.oo.ooo.oooooooooooooooooooooo

ooooooooooooooooooooooooooooooooooooooooo.oooooooo.o.o..ooo.

ooooooooo..oooo.ooooooooooooooooooo.oooooo.oooooooooooooooooo

oooooooooooooooooooooooooooooo.oo.oo.ooooooooooooooooooooooo

oooooooooooooooooooooooooooooooooooooooooooooooooooooooooooo

oooooo.oo.ooo.oo.oo.ooooooooooooooooooo.oooo.ooooooo.o.oo.oo

oooooooooooooooooooooooooo.oooooooooooooooooooooooo.o.ooo.oo

oooooooooooooo.oooo.oooooooooooooooooo.ooooo.oooooo.oo.ooooo

ooooooo.ooooooooooo.oooooooooooooo.ooo.ooo..ooooo.ooo.oooooo

..ooooooooooooooooooooo.oooooooooooooooo

"o" => good "%" => ok "!" => bad rmsd

"O" => overloaded "#" => many bad "." => weak

"@" => abandoned

Mosaic spread: 0.336 < 0.336 < 0.336

-------------------- Integrating SWEEP2 --------------------

Processed batches 2 to 1091

Standard Deviation in pixel range: 0.02 0.88

Integration status per image (60/record):

oo.oooooooooooo..o.oooooooo.ooooooooooooooooo.ooooo.oo.oooo.

ooooooooooooooooo.oooooo.oo.oo.ooooo.oooooooooooooooo.oooooo

ooooooooooooooo.oooooooooo.ooooooo.ooooooooooo.oo.oo.ooooooo

oooooo.ooo.oooooooooooo.oooooooooooooooooo.ooooooooooo..oo.o

ooo.ooooooo..oooooooooooooo.oooo.oo.ooooo.ooo.oo.ooooooooo.o

ooo...oo..oooooooo.oooooooooo.ooo.o.o.oooooooo.oo.ooo.oooooo

.ooooooooo.o.oooooooo.o.o..oooooooooo.o.ooooooooooooooo.o.o

...o.ooooooooooooooooooo.o.ooooooooooooooooooo.ooo.ooooooooo

..oooooooo.o.ooooo.o.oooooooooooooooooooooo.oooo.oooo.o.oooo

ooooooooooooooooooooooo.ooooooo.oooooooooooooooooo.oo.ooo.oo.

oooo.oooooooooooooooo.oooooooooooooooooooooooooooooo.oo..ooo

ooo.oo.oooooooooooo.ooooooooooooooooo.oo.oooo.ooooooo.o..ooo

..oo.oooooooooo.o.o.ooooo.oooooooooooooooooooooooooo.ooo.ooo

```
o.oo.ooooooooo.oooooooooo.oooooo.oooooooooooooo.oooooooooooo
oooooooooooooooooooooo.ooo.oooooooooooooooooo..ooooooooooo.oo
oooooooooooooooo.ooo.ooooooooooooooooooooooooo.oo.oooooooooo
oooooooo.ooooooooooo.oooo.ooooooo.oooo.ooooooo..oooooooooooo
ooooooooooo.o.oooooooooooooooo..ooooooo.oooooooooooooooooooo
oooooooooo
"o" => good        "%" => ok        "!" => bad rmsd
"O" => overloaded  "#" => many bad  "." => weak
"@" => abandoned
Mosaic spread: 0.264 < 0.264 < 0.264
-------------------- Integrating SWEEP3 --------------------
Processed batches 2 to 1031
Standard Deviation in pixel range: 0.01 2.20
Integration status per image (60/record):
ooooooo..o.oooooooooo.oo.ooooo.o.ooo.oo.oooooooooooooooo.ooo
oo.oooo.ooo.o...ooooooooo.%oo.ooooo.ooooooooooooo.oooo.oooooo
oooo..oo.oooooooooooooo.oo..ooooooo.oooo.oooo.o.ooo.oo.oooo.
ooooo.oooo.o.oooooooooooo.oooo.oooooooooo.oooo.oooo.ooooo.oo
ooooooo.oooooo.ooo..ooooo.oo.oooo.ooooo.oo.ooooooooooo..oooo
o.oooooooooooo.%.oooooooooooo.oo.oo.oo.o..oooooooooooooo..o.
..ooo.ooo.ooooooo.ooooo.ooo.o.o..o..oooooooo.oo.ooooo.oo.ooo
.o.ooooo..o.ooo..oooooooo..oooo.oooooooooo.ooooooo.oooooooo.
..ooo.oooooo.ooooooooo.oo.oooooo.oooooooooo.ooooooo.oo.oo.o.
oo..oooooooooooooo..oooo.ooooooooo..oooooooooo.ooo.o.ooooooo
oooooooo..o...ooo...oooooo.%o.oooo.oooooooooo.ooooooo.oo.o..
ooo.o.oooooooo.oo.o.oo.ooooooooo..o.oooooooooooooooo.ooooo.o.
ooo.oooooooooooooooo.oo..o..ooo.oooo.oo%oooo%.%oo!o.oooooooo
oooooooooooo%o%.o%ooooooooooooooo.o.oooo%ooooooooooooo.o.ooo
o.oooo.ooo.%oooo.oo.o.oooo..o.oo..ooooooooo.o.oooooo.ooo..o.
oo..oooooooo.oo.o.ooo.oooooooooooooooooooo%o.ooo.ooooooooooo
o.o.ooo.ooooooooooooo%.oooooooooooooooo.o.ooo.oooo.ooooooooo
ooooooooo.
"o" => good        "%" => ok        "!" => bad rmsd
"O" => overloaded  "#" => many bad  "." => weak
"@" => abandoned
Mosaic spread: 0.154 < 0.154 < 0.154
-------------------- Integrating SWEEP4 --------------------
Processed batches 2 to 641
Standard Deviation in pixel range: 0.01 1.28
Integration status per image (60/record):
ooooo..ooooooooooooooooooooooooooooooo.ooooooo.oooooooo.ooo.o
ooooooooooo.oooooooooo.ooooooooooooooooo.oooooo.oooooooooooo
oooooooooooooooooo.o.ooooo.ooo.oooooooooooooooooooo.oooo.ooo
ooooooooooo.ooooooo.ooooooooooooooooooooooooooooooooo.oooooooo
oooooooo.ooooooooo.ooooooooooooooooooooooooooooooooooo.o..oo
oooooooooooooooooooooo.ooooooooooooooooooooooo.oo.oooooooooo
.oooooooooooooooooooooooooooooooooooooooooooooooooooo..ooooo
oooooooooooo..oooooooooooooooooooooooooooo.oooooooooooooooo
o.o..ooooooooo.ooo.oooooooooooo.ooooooo.oooooooo.ooo.o.ooooo
ooooooo.o.oo.ooooooo.ooooooooooo.ooooooo%o%oooooooooooo.oooo
```

o..o%ooooo.o.ooooooo.oo.oo%ooooooooooo.o
"o" => good "%" => ok "!" => bad rmsd
"O" => overloaded "#" => many bad "." => weak
"@" => abandoned
Mosaic spread: 0.153 < 0.153 < 0.153
-------------------- Integrating SWEEP5 --------------------
Processed batches 2 to 641
Standard Deviation in pixel range: 0.05 1.19
Integration status per image (60/record):
o.ooooooo.oooooooooooo.ooooooooooooo.oooo.oo.oooo.ooooooo.oo
o.ooooooooo.ooooooooooo.ooo.o.oooo.ooooo.ooooooooooooooooooo
.oooo.ooo.oooooooooooo.o.oooooooooooooooooooooooooooooooo.oo
.oooo.ooooooooooooooooo.ooo.ooooooooooooo.oooooooo.o.oooo.oo
oooo.oo.oooooooooooo.oooo.ooooo..oooooooooooooooooooooooo.oo
oooooo.ooooo.ooo%oooo%ooooooooo.ooooooooooo.oooooooooooooooo
%oooooooooooooo.ooooooo..oooooooooooooo.oooooo.ooooooooooooo
ooooooooo.oooooo%o.oooooooooooooooooo.oooooooooooooooooooo.
oooooooooooooooooooo.ooooooooo.oooo.oooooooooooooooooooo.ooo
oo.ooo.oooo.ooooo.o..ooooooooooooooooooo.oo.oooooooo.o..o.o.
oooooooooooooooooooooooooo.oooo.oooooooo
"o" => good "%" => ok "!" => bad rmsd
"O" => overloaded "#" => many bad "." => weak
"@" => abandoned
Mosaic spread: 0.172 < 0.172 < 0.172
-------------------- Preparing DEFAULT ---------------------
Reindexing all datasets to common reference
--------------------- Scaling DEFAULT ----------------------
Resolution limit for NATIVE: 0.67 (user provided)
Resolution limit for NATIVE: 0.67 (user provided)
Resolution limit for NATIVE: 0.67 (user provided)
Resolution limit for NATIVE: 0.67 (user provided)
Resolution limit for NATIVE: 0.67 (user provided)
------------------- Unit cell refinement -------------------
Overall: 7.52 11.48 7.99 90.00 101.50 90.00
Project: AUTOMATIC
Crystal: DEFAULT
Sequence:
Wavelength name: NATIVE
Wavelength 0.53400
Sweeps:
SWEEP SWEEP1 [WAVELENGTH NATIVE]
TEMPLATE dmu35dnba_01_#####.cbf
DIRECTORY /dls/i19-2/data/2018/cm19670-5/20181210/07_dmu35dnba02
IMAGES (USER) 1 to 1240
MTZ file: /dls/i19-2/data/2018/cm19670-
5/processing/xia2GUI/07_dmu35dnba02_20201112_1136/DEFAULT/NATIVE/SWEEP1/integrate/29_integrated.refl

SWEEP SWEEP2 [WAVELENGTH NATIVE]
TEMPLATE dmu35dnba_02_#####.cbf
DIRECTORY /dls/i19-2/data/2018/cm19670-5/20181210/07_dmu35dnba02

IMAGES (USER) 1 to 1090
MTZ file: /dls/i19-2/data/2018/cm19670-5/processing/xia2GUI/07_dmu35dnba02_20201112_1136/DEFAULT/NATIVE/SWEEP2/integrate/31_integrated.refl

SWEEP SWEEP3 [WAVELENGTH NATIVE]
TEMPLATE dmu35dnba_03_#####.cbf
DIRECTORY /dls/i19-2/data/2018/cm19670-5/20181210/07_dmu35dnba02
IMAGES (USER) 1 to 1030
MTZ file: /dls/i19-2/data/2018/cm19670-5/processing/xia2GUI/07_dmu35dnba02_20201112_1136/DEFAULT/NATIVE/SWEEP3/integrate/33_integrated.refl

SWEEP SWEEP4 [WAVELENGTH NATIVE]
TEMPLATE dmu35dnba_04_#####.cbf
DIRECTORY /dls/i19-2/data/2018/cm19670-5/20181210/07_dmu35dnba02
IMAGES (USER) 1 to 640
MTZ file: /dls/i19-2/data/2018/cm19670-5/processing/xia2GUI/07_dmu35dnba02_20201112_1136/DEFAULT/NATIVE/SWEEP4/integrate/35_integrated.refl

SWEEP SWEEP5 [WAVELENGTH NATIVE]
TEMPLATE dmu35dnba_05_#####.cbf
DIRECTORY /dls/i19-2/data/2018/cm19670-5/20181210/07_dmu35dnba02
IMAGES (USER) 1 to 640
MTZ file: /dls/i19-2/data/2018/cm19670-5/processing/xia2GUI/07_dmu35dnba02_20201112_1136/DEFAULT/NATIVE/SWEEP5/integrate/37_integrated.refl

| For AUTOMATIC/DEFAULT/NATIVE | Overall | Low | High |
|---|---|---|---|
| High resolution limit | 0.67 | 1.81 | 0.67 |
| Low resolution limit | 11.48 | 11.48 | 0.68 |
| Completeness | 97.5 | 100.0 | 94.4 |
| Multiplicity | 4.8 | 6.4 | 3.5 |
| I/sigma | 25.8 | 197.4 | 1.0 |
| Rmerge(I) | 0.025 | 0.009 | 0.360 |
| Rmeas(I) | 0.028 | 0.010 | 0.426 |
| Rpim(I) | 0.012 | 0.004 | 0.217 |
| CC half | 1.000 | 1.000 | 0.551 |
| Total observations | 11755 | 887 | 415 |
| Total unique | 2424 | 139 | 117 |

Assuming spacegroup: P 1 2/m 1
Unit cell (with estimated std devs):
7.5196(8) 11.4783(8) 7.9857(9)
90.0 101.498(10) 90.0
mtz_unmerged format:
Scaled reflections (NATIVE): /dls/i19-2/data/2018/cm19670-5/processing/xia2GUI/07_dmu35dnba02_20201112_1136/DataFiles/AUTOMATIC_DEFAULT_scaled_unmerged.mtz
mtz format:
Scaled reflections: /dls/i19-2/data/2018/cm19670-5/processing/xia2GUI/07_dmu35dnba02_20201112_1136/DataFiles/AUTOMATIC_DEFAULT_scaled.mtz
Processing took 00h 04m 48s
XIA2 used... dials, dials.scale, xia2
Here are the appropriate citations (BIBTeX in xia2-citations.bib.)
Beilsten-Edmands, J. et al. (2020) Acta Cryst. D76.

| |
|---|
| Winter, G. (2010) J. Appl. Cryst. 43, 186-190.<br>Winter, G. et al. (2018) Acta Cryst. D74, 85-97.<br>Status: normal termination<br><br>END OF FILE |

**Table S3** Commands used alongside xia2 (Winter, 2010) with DIALs (Winter *et al.*, 2018) for performing data reduction for SQABPY-I case study.

| |
|---|
| xia2 /dls/i19-2/data/2019/cm22964-3/ELF_20190808/022_SQABPY_0V small_molecule=True d_min=0.75 unit_cell=3.79,11.21,27.45,90,92.24,90 space_group=P21/n |

**Table S4** Hydrogen atom treatment during crystal structure solution and refinement for the case study SQABPY-I before (yellow form), during high voltage (red-shifted yellow form) and after (yellow form) voltage off including details of the proton disorder model.

| |
|---|
| Hydrogen atoms were located in the Fourier difference map. In the case of the during high voltage form, the disordered H-atom (H1 and H1A) was modelled on two sites (N1 and O1) using the PART and FVAR instruction, a DANG restraint for C12-H1 and C13-H1 and isotropic thermal parameters H1 and H1A were constrained to be equal using the EADP instruction.) |

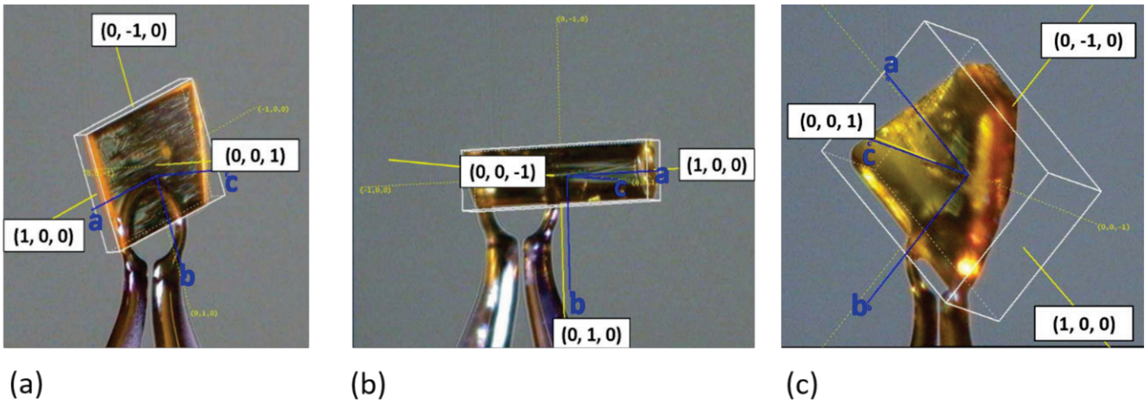


**Figure S2** Face indexing of additional single crystals of SQABPY-I (a – c) showing crystal faces (yellow lines perpendicular to the white box faces) and unit cell axes (blue).

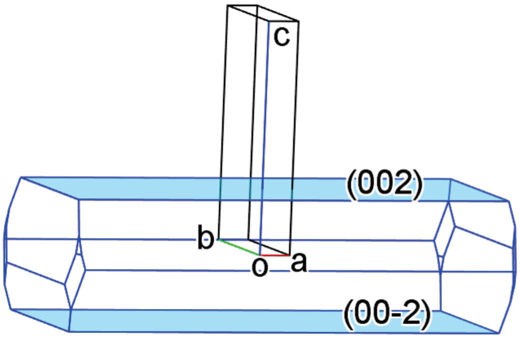


**Figure S3** BFDH Mercury™ (Macrae *et al.*, 2006, Macrae *et al.*, 2020) crystal morphology prediction for SQABPY-I, predicting that the a-axis coincides with the long crystal length as found during face indexing and that 002 and 00-2 are the dominant growth faces (the crystal width) and are perpendicular to the c-axis.

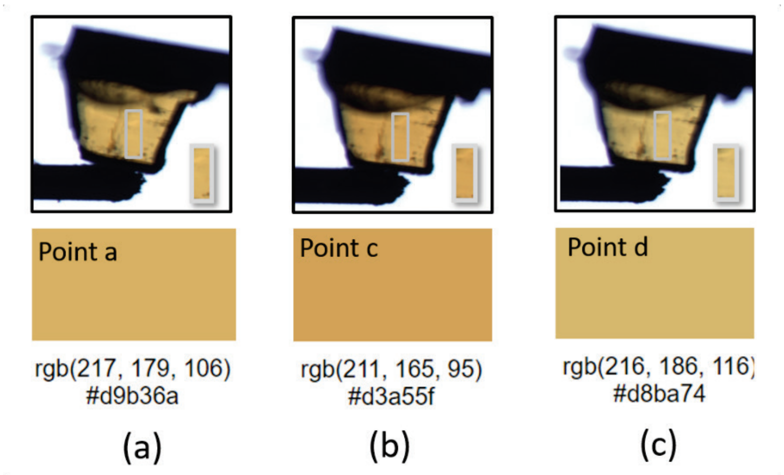


**Figure S4** Average colour picker (Matkl@github, Accessed 02/06/2021) from selected area (grey box) of offline voltage application images (manuscript, Figure 8) at (a) point a: before voltage 0 V, (b) point c: during voltage 1900 V and (c) point d: after voltage 0V. Rgb and hex (#) codes are included for each average colour.

**Table S5** The unit cell parameters as a function voltage for the before (yellow form), during high voltage (red-shifted yellow form) and after (yellow form) voltage off:

| | Voltage (V) | *a* (Å) | *b* (Å) | *c* (Å) | *β* (°) | Volume (Å$^3$) |
|---|---|---|---|---|---|---|
| Before | 0 | 3.80063(14) | 11.2125(3) | 27.4464(7) | 92.272(3) | 1168.69(5) |
| During | 2400 | 3.8006(2) | 11.2165(5) | 27.4621(11) | 92.271(11) | 1169.77(9) |
| After | 0 | 3.7999(3) | 11.2238(6) | 27.4932(14) | 92.277(6) | 1171.64(13) |

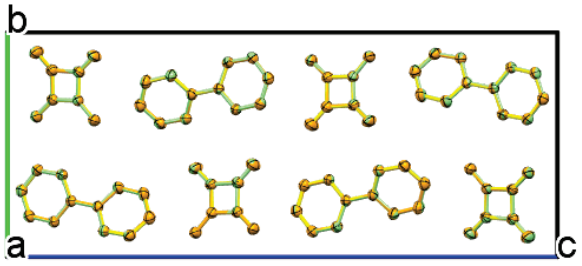


Figure S5 Overlaid crystal structures of the before voltage (0 V) yellow form (coloured yellow), the during high voltage (2400 V) red-shifted yellow form (coloured orange) and the after voltage off (0 V) yellow form (coloured green).
